# The relationship between social support, cognitive function and activities of daily living in older people: a cross-sectional study

**DOI:** 10.3389/fpubh.2025.1602466

**Published:** 2025-06-25

**Authors:** Man Feng, Jinxiu Li, Fen Xie, Zhengying Chen

**Affiliations:** ^1^Longgang Central Hospital of Shenzhen, Shenzhen, China; ^2^Jishou University School of Medicine, Jishou, China; ^3^School of Nursing and Midwifery, Trinity College Dublin, Dublin, Ireland

**Keywords:** older people, social support, cognitive function, activities of daily living, cross-sectional study

## Abstract

**Introduction:**

With the increasing severity of population aging, the prevalence of chronic diseases and disabilities is rising, significantly impacting older people’s activities of daily living (ADL), and overall quality of life. Social support plays a crucial role in maintaining their health, as higher levels of support are associated with better quality of life, while inadequate support can negatively affect cognitive function, and ADL. Therefore, this study aimed to investigate the relationship between social support, cognitive function, and activities of daily living (ADL) in older people.

**Methods:**

Between 2022 and 2023, 1,600 older people were selected for the survey using a multi-stage stratified random cluster sampling method. Participants completed a questionnaire regarding demographics, social support, mini-mental state examination, and ADL. Pearson correlation analysis was used to explore the associations among social support, cognitive function, and ADL.

**Results:**

The results showed that 81.4% of older people had a medium level of social support, 53.1% had cognitive impairment, and 28.3% had impaired ADL. There was a positive correlation between social support and cognitive function (*r* = 0.168, *p* < 0.001), a negative correlation between social support and ADL (*r* = −0.269, *p* < 0.001); and a negative correlation between cognitive function and ADL (*r* = −0.142, *p* < 0.001).

**Conclusion:**

The social support of older people was at a medium level, the cognitive function level was low, and the rate of impairment of ADL was high. There was a significant correlation between social support, cognitive function, and ADL.

## 1 Introduction

The World Health Organization reports that the number of people aged 60 and over globally was 1 billion in 2019 and will increase to 2.1 billion in 2050 (1). The increasing longevity has enabled more and more older people to enjoy extra years of life, improving overall human well-being (2). However, the health challenges brought about by population aging have become increasingly prominent, and the prevalence of chronic diseases, cognitive impairment, and disability has gradually increased (3–5). With aging, cognitive functions inevitably decline, often manifested as declining memory, reduced attention span, and weakened language abilities. If this deterioration progresses further, it may lead to dementia. This not only significantly impairing older people’s independence in activities of daily living (ADL) (6), and seriously impacts their quality of life (7), but also imposes significant psychological stress on both older individuals and their families (8). Furthermore, it substantially increases the economic and social burdens associated with aging-related health issues (9, 10).

In this context, social support, as a positive social resource, plays a key role in maintaining the health and functional independence of older adults. Social support refers to a series of beneficial assistance and support that individual receive in social interactions, including emotional support, instrumental support, and informational support. It aims to enhance individuals’ ability to manage stress, and help individuals better cope with difficulties and challenges in life, thereby maintaining a positive attitude and reducing the negative impact of psychological problems on their lives (11). Research indicates that older people with higher levels of social support generally experience better quality of life and greater life satisfaction (12). Individuals who receive more social support tend to exhibit better overall cognitive function (13). Compared to older people with low levels of leisure activity participation, those who engage in high levels of leisure activity had a significantly lower risk of cognitive impairment (14). In addition, studies have found that social support interventions can alleviate the deterioration of ADL function. Good social connections can provide practical help and psychological support for older people, thereby reducing the incidence of ADL disorders (15, 16). Conversely, inadequate social support may negatively affect cognitive function, emotional well-being, and ADL (12). Older people with poor social support have relatively poor cognitive functions, which leads to physical and mental damage and disability. While some ADL limitations can be delayed (17), disability often leads to a range of adverse consequences, including significantly higher healthcare costs (18), increased mortality (19), and reduced quality of life (20). Therefore, it is essential for older people to build strong social networks, enhance social support, improve cognitive function, and reduce ADL impairments to promote their overall health and well-being.

However, most current studies tend to explore the effects of social support on cognitive function and ADL separately, and few studies systematically analyze the potential relationship between the three. Therefore, this study aims to explore the current status of social support, cognitive function, and ADL in older people, and to deeply analyze the correlation between the three, in order to provide scientific basis and practical guidance for improving the overall health level and quality of life of older people.

## 2 Methods

### 2.1 Design

A cross-sectional study was conducted during 2022–2023. The study was reported following the Strengthening the Reporting of Observational Studies in Epidemiology (STROBE) checklist.

### 2.2 Study setting and sampling

The sample size was calculated using the formula *N* = (UαS/*δ*)^2^. Based on the preliminary survey results, the standard deviation S of older people’s ADL was 4.758. The test level *α* was set to 0.05, and the allowable error *δ* was set to 0.238. Upon substituting these values into the formula, the calculated required sample size was 1,535. Additionally, considering a potential 10% sample loss rate, the sample size was expanded to 1,688.

A multi-stage stratified random cluster sampling method was used. First, a county was randomly selected from the Wuling Mountain area of China (Hunan Province, Hubei Province, Guizhou Province, and Chongqing Municipality). Second, a street was randomly selected from each of the selected counties. Finally, two neighborhood committees were randomly selected from the selected streets. All eligible older people in these two neighborhood committees were included in the study. If the number of eligible older people in the selected neighborhood committee was small, another neighborhood committee in the street to which it belonged was selected to supplement the sample until the sample size was reached.

### 2.3 Inclusion and exclusion criteria

Inclusion criteria: (1) age ≥ 60 years; (2) able to complete the survey independently or with the assistance of data collectors; (3) informed consent and willingness to participate in this study. Exclusion criteria: (1) people with unconsciousness or mental disorders; (2) people with serious diseases and terminal diseases.

### 2.4 Research tools

Research tools include demographic, social support rate scale, mini-mental state examination scale and activities of daily living scale.

#### 2.4.1 Demographic

Participants provided information on their age, gender, nationality, marital status, education level, place of residence, monthly living allowance for children (yuan), chronic disease (self-reported), smoking, and drinking.

#### 2.4.2 Social support rate scale (SSRS)

The SSRS was created by Xiao and Yang (21). This scale has strong retest reliability (*r* = 0.92), with high reliability and validity across all items (Cronbach’s *α* coefficients ranging from 0.89 to 0.94). The scale consists of 10 items, covering three dimensions: subjective support, objective support, and support utilization. The total score ranges from 12 to 66 points. The higher the score, the more support the individual receives. Specifically, a total score of 22 or below is categorized as a low level of social support, scores ranging from 23 to 44 indicate a moderate level, and scores between 45 and 66 represent a high level of social support.

#### 2.4.3 Mini-mental state examination (MMSE)

The MMSE scale was originally developed by Folstein et al. (22) in 1975 and is used to quickly assess an individual’s cognitive function. In 1988, Li et al. (23) revised the MMSE scale for Chinese and localization, and its test–retest reliability was 0.88. The MMSE scale contains 30 items covering five main dimensions: orientation, registration, attention and calculation, recall, and language. The total score ranges from 0 to 30 points. The MMSE score results are affected by education level, and the criteria for cognitive impairment were illiterate ≤ 17 points, primary school ≤ 20 points, and junior high school and above ≤ 24 points.

#### 2.4.4 Activities of daily living scale (ADLs)

ADLs consists of two parts: physical self-maintenance and instrumental activities of daily living, with a total of 14 items (24). The Cronbach’s *α* coefficient of this scale was 0.811. The scale uses a 4-level scoring system, with the lowest total score being 14 points, representing normal ADL; if the total score exceeds 14 points, it indicates that there are varying degrees of functional impairment in ADL. Specifically, 15–28 points were considered mild functional impairment, 29–42 points were considered moderate functional impairment, and 43–56 points were considered severe functional impairment.

### 2.5 Data collection

Before data collection, all data collectors received unified training to ensure that they had a clear understanding of the purpose and significance of the study, mastered the correct method of filling out the questionnaire, avoided using misleading language, and ensured the accuracy of the data. To protect the privacy and confidentiality of the participants, the questionnaires were collected anonymously. Paper questionnaires were distributed to participants to be filled in by themselves. If participants were unable to fill in the questionnaires themselves, the data collectors read the questionnaires item by item to the participants, and they answered the questions themselves after understanding them, and the data collectors helped them fill in the questionnaires. A total of 1,680 questionnaires were distributed, and 1,660 were collected, with a questionnaire recovery rate of 98.8%. After excluding 60 invalid questionnaires, the questionnaire validity rate was 95.2%.

### 2.6 Data analysis

SPSS 25.0 was used for data analysis. Descriptive statistics were used to summarize the demographic characteristics of older people. Pearson correlation analysis was used to explore the correlation between social support, cognitive function, and ADL. *P* < 0.05 was considered statistically significant.

### 2.7 Ethical considerations

This study was approved by the Biomedical Ethics Committee of Jishou University. Before participating in the study, each participant was informed in detail about the purpose, importance, and relevant ethical considerations of this study. All participants participated voluntarily and provided written consent. Participants were clearly informed that they were free to withdraw at any stage during the study without giving a reason and that their decision would not affect any medical or nursing services they received. All participants participated in this study mainly out of concern for their own health and willingness to support the study. This study did not provide any form of financial or material subsidies to the participants.

## 3 Results

### 3.1 Demographics and characteristics of older people

This study included 1,600 older people with a mean age of 75.5 ± 7.8 years. The majority were male (50.3%), minority nationality (69%), and married (71%). 60.0% of older people live in urban, and 66.3% have three or more children. It is worth noting that 90% of older people suffer from chronic diseases, of which 60.5% suffer from three or more chronic diseases. See Table 1 for details.

**Table 1:** Demographics and characteristics of older people (*N* = 1,600).

Variable	Characteristic	*N*	%
Age	60–69	503	31.4
	70–79	782	48.9
	80–89	270	16.9
	≥90	45	2.8
Gender	Male	805	50.3
	Female	795	49.7
Nationality	Han	496	31.0
	Minority nationality	1,104	69.0
Marital status	Single	32	2.0
	Married	1,136	71.0
	Divorced	112	7.0
	Widowed	320	20.0
Education level	Illiteracy	480	30.0
	Primary school	704	44.0
	Junior high school	328	20.5
	Senior high school/technical secondary school	64	4.0
	College/undergraduate and above	24	1.5
Place of residence	Urban	960	60.0
	Rural	640	40.0
Number of children	0	32	2.0
	1–2	508	31.8
	≥3	1,060	66.3
Monthly living allowance for children (yuan)	0	480	30.0
	≤1,000	480	30.0
	1,000–1999	528	33.0
	2000–3,999	112	7.0
	Retirement and pension	504	31.5
Chronic disease	Yes	1,440	90.0
	No	160	10.0
Smoking	Yes	757	47.3
	No	843	52.7
Drinking	Yes	743	46.4
	No	857	53.6

### 3.2 Current status of social support, cognitive function, and activities of daily living in older people

The results showed that the level of social support, cognitive ability, and total ADL score were 32.97 ± 8.04, 19.87 ± 4.61, and 17, respectively. Most older people had a medium level of social support, accounting for 81.4% (*n* = 1,303), 53.1% had cognitive impairment (*n* = 849), and 28.3% had disabilities (*n* = 453). See Table 2.

**Table 2:** Current status of social support, cognitive function, and activities of daily in older people.

Variable	Characteristic	*N*	%
Social support	Low level	159	9.9
	Medium level	1,303	81.4
	High level	138	8.6
Cognitive function	Normal	751	46.9
	Cognitive impairment	849	53.1
Activities of daily living	Normal	1,147	71.7
	Disability	453	28.3

### 3.3 Correlation analysis among social support, cognitive function and ADL in older people

The results showed that the total score of cognitive function was positively correlated with the total score of social support (*r* = 0.168, *p* < 0.001). The dimensions of cognitive function orientation, recall, and language were positively correlated with the total score and sub-dimensions of social support (*p* < 0.001). It is worth noting that the registration dimension of cognitive function was negatively correlated with the support utilization dimension of social support (*r* = −0.074, *p* < 0.01). The reason for this result may be that some older people with cognitive decline are more likely to rely frequently on external support resources, or that they exhibit more dependent behaviors when faced with cognitive decline. See Table 3.

**Table 3:** Correlation analysis between social support and cognitive function in older people.

Variable	Cognitive function	Orientation	Registration	Attention and calculation	Recall	Language
Social support	0.168^***^	0.125^***^	−0.001	0.142^***^	0.164^***^	0.182^***^
Subjective support	0.237^***^	0.180^***^	0.046	0.204^***^	0.171^***^	0.258^***^
Objective support	0.030^***^	0.013^***^	−0.015	0.013	0.117^***^	0.017^***^
Support utilization	0.090^***^	0.078^***^	−0.074^**^	0.089^***^	0.099^***^	0.119^***^

**P < 0.01; ***p < 0.001.

The total score of social support was negatively correlated with the total score of ADL (*r* = −0.269, *p* < 0.001). The subjective support, objective support, and support utilization of social support were negatively correlated with the total score of ADL (*r* = −0.274, *p* < 0.001) and each sub-dimension (*p* < 0.001), see Table 4.

**Table 4:** Correlation analysis between social support and ADL in older people.

Variable	Social support	Subjective support	Objective support	Support utilization
Activities of daily living	−0.269^***^	−0.274^***^	−0.186^***^	−0.284^***^
Physical self-maintenance	−0.243^***^	−0.260^***^	−0.165^***^	−0.267^***^
Instrumental activity of daily living	−0.272^***^	−0.277^***^	−0.186^***^	−0.283^***^

***P < 0.001.

The total score of cognitive function was negatively correlated with the total score of ADL (*r* = −0.142, *p* < 0.001). The cognitive function dimensions of orientation, registration, attention and calculation, and language were negatively correlated with the total score and sub-dimensions of ADL (*p* < 0.001). In addition, the recall dimension of cognitive function was negatively correlated with the physical self-maintenance dimension (*r* = −0.054, *p* < 0.05), see Table 5.

**Table 5:** Correlation analysis between cognitive function and ADL in older people.

Variable	Cognitive function	Orientation	Registration	Attention and calculation	Recall	Language
Activities of daily living	−0.142^***^	−0.121^***^	−0.117^***^	−0.131^***^	−0.040	−0.148^***^
Physical self-maintenance	−0.145^***^	−0.118^***^	−0.106^***^	−0.136^***^	−0.054^*^	−0.151^***^
Instrumental activity of daily living	−0.134^***^	−0.119^***^	−0.114^***^	−0.124^***^	−0.035	−0.141^***^

*P < 0.05; **P < 0.0; ***P < 0.001.

## 4 Discussion

The results of this study showed that the total score of social support was 32.97 ± 8.04, which was lower than the Chinese norm (34.56 ± 3.73) (21). Overall, it was at a medium level (81.4%). The rate of cognitive impairment was 53.1%, higher than that in China (15.5%) (25) and Shenyang (33.26%) (26). The rate of ADL impairment was 28.30%, higher than that in Kunshan, Jiangsu (15.50%) (27). This may be related to the unique geographical location, eating habits, ethnic culture, lifestyle, economic conditions, and medical service levels of the Wuling Mountain area.

There was a significant positive correlation between social support and cognitive function in older people. Specifically, older people with higher levels of social support also have higher levels of cognitive function. This finding was supported by the study by Zhu et al. (14), which showed a significant association between active participation in leisure activities and a lower risk of cognitive impairment. The reason is that physical exercise can promote positive physiological and metabolic changes, such as increased levels of brain-derived neurotrophic factor, enlarged hippocampal volume, and improved functional adaptability. These physiological effects collectively enhance memory function and significantly lower the risk of cognitive impairment in older adults (28). When the cognitive functions of older people are enhanced, their negative emotions tend to be alleviated, and they experience higher happiness and satisfaction at the cognitive level (29). In addition, previous studies have shown that educational level can affect the ability to participate in social activities and performance on cognitive tests (30), potentially interfering with research results. Yun et al. (30) surveyed older people in Suzhou communities and found that the prevalence of cognitive impairment was 32.0%, significantly lower than the 53.1% in this study. This difference may be related to the different proportions of people with high education in the sample. In Yun’s study, the proportion of people with high school education or above reached 50% (30), while the proportion in this study was only 5.5%. In addition, social support is also believed to have a potential positive role in the prevention of chronic diseases and health maintenance. Cofie et al. (31) found that social support in the form of emotional support, health guidance, and information exchange was associated with a lower risk of chronic diseases (such as hypertension and diabetes).

There was a significant negative correlation between social support and ADL impairment, which aligns with the findings of Van Orden et al. (32). Higher levels of social support were associated with a lower likelihood of ADL impairment. Feng (33) also confirmed this finding. There was a positive correlation between the level of social support and the self-care ability of older people. That is, the higher the level of social support of older people, the stronger their ADL, and the lower the risk of developing ADL impairment. In addition. Those with high social support tend to maintain a positive attitude and exhibit greater optimism toward life (34). Positive social interactions and high levels of social support play a crucial role in fostering the development of positive personality traits in older people (35, 36). Older people with high social support are often cheerful, open-minded, and maintain extensive and harmonious interpersonal relationships. They are more willing to go out of the house, actively engage in social activities, and maintain close communication with relatives and friends, which significantly enriches their daily lives. When faced with negative emotions such as loneliness or depression, older people with high social support can more easily obtain emotional comfort and assistance from friends and family. This support not only helps them cope more effectively with life’s difficulties and challenges, but it also motivates them to actively participate in health-promoting activities, such as physical exercise.

There was a significant negative correlation between cognitive function and ADL, which is consistent with the results of Edwards et al. (37). Specifically, older people with lower cognitive function are more likely to experience a higher degree of ADL impairment. Song (38) further discovered that cognitive impairment of older people is associated with physical frailty, and that cognitive impairment can further lead to ADL impairment. When older people experience a decline in their ADL, their range of activities tends to shrink, leading to reduced interaction with the outside world and limited access to information. This isolation may increase the risk of disuse syndrome, which negatively impacts cognitive function and can result in an overall decline in cognitive abilities.

## 5 Limitations

The study has several limitations. First, it surveyed only 1,600 older people in the Wuling Mountain area, limiting the representativeness of the sample and potentially introducing bias into the findings. Second, as the study was a cross-sectional survey and lacked longitudinal data support, it was impossible to investigate the causal mechanisms underlying the observed relationships. In addition, some participants with poor cognitive function received assistance from data collectors when filling out the questionnaires, which may have introduced subjective judgments and affected objectivity. Future research should expand the sample size to include older adults from different geographical and cultural backgrounds, and adopt longitudinal tracking to explore the causal mechanisms among variables. At the same time, the specific roles and intervention effects of different forms of social support should be further analyzed, develop a more robust older adults health assessment system and design more effective and personalized intervention strategies.

## 6 Conclusion

The results of this study show that the social support level of older people is at a medium level and still needs to be further improved. At the same time, the cognitive function level is low and the ADL impairment rate is high. Further analysis found that there was a positive correlation between social support of older people and cognitive function, and a negative correlation with ADL. A negative correlation was found between ADL and cognitive function. Based on this, further attention should be paid to the cognitive function and ADL status of older people in the future, and the development of a multi-level social support system should be encouraged. For example, at the policy level, consideration can be given to increasing investment in cognitive health screening and precision health interventions for older people, such as personalized training for early cognitive decline. At the community level, the establishment of intergenerational interaction projects and senior activity centers can be promoted to enhance older people’s sense of social participation. At the family level, caregivers’ education and psychological support can be strengthened, and emotional and instrumental support within the family can be enhanced.

## Data Availability

The original contributions presented in the study are included in the article/supplementary material, further inquiries can be directed to the corresponding author/s.
